# Intestinal mucositis precedes dysbiosis in a mouse model for pelvic irradiation

**DOI:** 10.1038/s43705-021-00024-0

**Published:** 2021-06-10

**Authors:** Charlotte Segers, Mohamed Mysara, Jürgen Claesen, Sarah Baatout, Natalie Leys, Sarah Lebeer, Mieke Verslegers, Felice Mastroleo

**Affiliations:** 1 Interdisciplinary Biosciences group, Belgian Nuclear Research Centre SCK CEN, Mol, Belgium; 2 Department of Bioscience Engineering, University of Antwerp, Antwerp, Belgium; 3 Department of Epidemiology and Data Science, Amsterdam UMC, VU University Amsterdam, Amsterdam, The Netherlands; 4 Department of Biotechnology, University of Ghent, Ghent, Belgium

**Keywords:** Intestinal diseases, Microbiome, Biomarkers

## Abstract

Pelvic radiotherapy is known to evoke intestinal mucositis and dysbiosis. Currently, there are no effective therapies available to mitigate these injuries, which is partly due to a lack of insight into the events causing mucositis and dysbiosis. Here, the complex interplay between the murine host and its microbiome following pelvic irradiation was mapped by characterizing intestinal mucositis along with extensive 16S microbial profiling. We demonstrated important morphological and inflammatory implications within one day after exposure, thereby impairing intestinal functionality and inducing translocation of intraluminal bacteria into mesenteric lymph nodes as innovatively quantified by flow cytometry. Concurrent 16S microbial profiling revealed a delayed impact of pelvic irradiation on beta diversity. Analysis of composition of microbiomes identified biomarkers for pelvic irradiation. Among them, members of the families *Ruminococcaceae*, *Lachnospiraceae* and *Porphyromonadaceae* were differentially affected. Altogether, our unprecedented findings showed how pelvic irradiation evoked structural and functional changes in the intestine, which secondarily resulted in a microbiome shift. Therefore, the presented in vivo irradiation-gut-microbiome platform allows further research into the pathobiology of pelvic irradiation-induced intestinal mucositis and resultant dysbiosis, as well as the exploration of mitigating treatments including drugs and food supplements.

## Introduction

Gut dysbiosis is generally defined as the qualitatively and quantitatively altered composition of intestinal microbiota, which offers an advantage for emergence and outbreak of pathogens and has a cascading impact on the immune system.^1^ In fact, dysbiosis is well known to develop following radiotherapy and was shown to be associated with the severity of irradiation-induced intestinal mucositis.^2,3^ In accordance, germ-free mice were reported to be more resistant to lethal (16 Gy) total body irradiation as compared to conventionally-housed specific pathogen-free mice.^4^ However, whether dysbiosis represents a cause or a consequence of irradiation-induced intestinal mucositis is not well understood.

Currently, there are no effective therapies available to mitigate irradiation-induced intestinal mucositis in human patients. Therefore, given their safe character, food supplements including probiotics, prebiotics, and vitamins are being investigated for their potential to provide radioprotection and re-establish an orthobiotic (i.e., balanced composition providing beneficial effects to the host) gut microbiome.^5^ For example, the probiotic preparation VSL#3 reduced the severity and incidence of irradiation-induced diarrhea in a Phase II clinical trial.^6^ The major limitation in the search for new food supplements is the lack of mechanistic understanding of the events underlying irradiation-induced intestinal mucositis and dysbiosis, as well as the lack of an appropriate research model system. For instance, cell (co-)culture set-ups based on intestinal epithelial cells are largely incomplete to capture the complexity of the intestinal tissue, the gut microbiome and the underlying pathobiology of irradiation-induced enteropathy.^7–10^ Additionally, total body irradiation is typically used as experimental modality, which however evokes a concomitant hematopoietic syndrome aggravating irradiation-induced mucositis.^11^ In this regard, the importance of the gut microbiome in the response to local pelvic irradiation has not been addressed in sufficient detail.^12–15^

In this study, we present novel insights in the structural and functional implications of intestinal mucositis and the consequent development of dysbiosis in a mouse model for pelvic irradiation by using an innovative multi-level approach. Within one day, pelvic irradiation initiated intestinal mucositis as shown by histology and hallmarks of inflammation. Then, a functionally disrupted barrier resulted in bacterial translocation investigated through the innovative use of flow cytometry. Consequently, extensive 16S microbial profiling revealed delayed changes in the gut microbiota composition. Our research thus presents an in vivo irradiation-gut-microbiome test platform to study the pathobiology and new potential treatments for pelvic irradiation-induced intestinal mucositis and secondary gut microbial dysbiosis.

## Material and methods

### Mice

All animals were housed at the specific pathogen free animal facility of SCK CEN in accordance with the Ethical Committee Animal Studies of Medanex Clinic (EC MxCl 2018-093) and were approved by the SCK CEN animal welfare committee. All animal experiments were carried out in compliance with the Belgian laboratory animal legislation and the European Communities Council Directive of 22 September 2010 (2010/63/EU).

Five weeks-old, male C57Bl/6JRj mice were purchased from Janvier (Bio Services, The Netherlands) and housed individually in ventilated cages under standard laboratory conditions (12‐hr light/dark cycle). Upon arrival, mice were acclimatized for two weeks. All animals had access to food and water *ad libitum* in the formulation of DietGel^®^ 76A (Bio Services, The Netherlands) supplemented with 10% maltodextrin (Applichem, Germany).

To minimize confounding factors across experimental cohorts, only male mice were used, housed in the same room and controlled for aging effects by choosing age-matched mice.^16^ In addition, potential other confounding factors including care taker(s), order of handling and differences in weight were investigated and excluded for its impact on the microbiome.

### Irradiation protocol

The effects of pelvic irradiation were studied by applying a sub-lethal dose of 12 Gy as previously described.^17,18^ Eight weeks-old mice were anesthetized by intraperitoneal injection of 50 mg/kg ketamine hydrochloride (Nimatek, Eurovet Animal Health, The Netherlands) and 0.25 mg/kg medetomidine hydrochloride (Domitor, Orion Corporation, Finland). To minimize distress caused by dried out eyes, a 2 mg/kg carbomerum gel (Vidisic, Bausch+Lomb, Belgium) was applied. A maximum of seven mice were placed in a custom-made disk-shaped Plexiglas box (20 cm diameter, 5 cm height), with each individual animal in a prone position and their lower body parts towards the center. The box was covered by a lead shield (5 mm thick) except for the center of the cover (9 cm diameter) allowing local, pelvic irradiation of the mice (Supplementary Fig. 1A). During transportation to the SCK CEN irradiation facility, mice were positioned in an incubator with a constant temperature range of 35–37 °C. Acute, single‐dose X‐irradiation (0 Gy or 12 Gy) was performed using an Xstrahl 320 kV tube (dose rate of 0.78 Gy/min; inherent filtration: 3 mm Be; additional filtration: 3.8 mm Al + 1.4 mm Cu + Dose Area Product monitor ionizing chamber; tube voltage: 250 kV; tube current: 12 mA; beam orientation: vertical) in accordance to ISO 4037 and under ISO 17025 accreditation. During the irradiation procedure, mice were placed on a heating plate set at 35–37 °C. To rule out effects induced by anesthesia or procedure-induced stress, control (0 Gy) mice were also anesthetized and transported to the irradiation facility but were not exposed to pelvic irradiation (i.e., sham-irradiation). After (sham-)irradiation, anesthesia was reversed by intraperitoneal injection of 1 mg/kg atipamezole hydrochloride (Antisedan, Orion Corporation, Finland) whilst positioned under a heating lamp.

During the entire experimental setup of maximum 14 days, mice were monitored on a daily basis including follow up of food intake and body weight. Eventually, all mice were sacrificed at post-irradiation day (PID) 1 (*n* = 4 per group), 3 (*n* = 7 per group) and 7 (*n* = 7 per group) (Supplementary Fig. 1B).

### Characterization of intestinal mucositis

Details on the procedures of histology, immunohistochemistry, intestinal myeloperoxidase activity assay and claudin 5 western blot are described in the supplementary information.

### Bacterial translocation to mesenteric lymph nodes

Mesenteric lymph nodes were aseptically resected out of the mesentery adjacent to the ileocecal valve and the ascending colon. They were collected in sterile, ice cold phosphate buffered saline and mashed manually using a 10 mL syringe plunger through a 40 µm nylon cell strainer (VWR, Belgium). Homogenized mesenteric lymph nodes were analyzed for total cell count by the innovative use of flow cytometry, as an alternative to bacterial plate counting. This allowed for a more complete bacterial counting by avoiding arbitrary growth selection based on (an-)aerobic cultivation or medium used. Briefly, samples were diluted (1:5 000; to obtain an event rate between 200 and 2 000 events/µl) in 0.2 µm filtered phosphate buffered saline, and additionally filtered using a 5 µm syringe filter to remove eukaryotic cells. Then, SYBR Green I dye (at a final concentration of 1:10 000; Invitrogen, USA) was added and samples were incubated for 20 min at 37 °C. Stained bacterial suspensions were then processed on a BD Accuri C6 flow cytometer (BD Biosciences, Belgium) and detected in the FL-1 channel (533/30 nm) after excitation with the blue laser (488 nm, 20 mW). The device was calibrated according to manufacturer’s instructions. Eventually, all samples were analyzed using the BD Accuri C6 software (v.1.0.264.21).

### Fecal DNA extraction and 16S rRNA gene sequencing

Fecal samples were longitudinally collected before, as well as one day, three days and seven days after exposure, to characterize acute (at PID1) and delayed (at PID3 and PID7) effects. At all sampling points, seven mice were included in each group. Total fecal DNA was extracted using the DNeasy PowerSoil Pro Kit (Qiagen, The Netherlands), according to manufacturer’s instructions. Extracted DNA concentrations were quantified by the QuantiFluor dsDNA system (Promega, the Netherlands). High-throughput amplicon sequencing of the V3-V4 hypervariable region was performed on BaseClear’s Illumina MiSeq (V3 chemistry) platform according to manufacturer’s guidelines. Herein, a positive control in the form of a mock DNA sample, and negative controls in the form of blank extraction and blank library samples were included as recommended.^19^

### Sequencing data processing and diversity analysis

Paired-end sequencing data were analyzed through the OCToPUS pipeline,^20^ consisting of pre-assembly correction (SPAdes tool, v.3.5.0,^21^) assembly, quality filtering and alignment (mothur, v.1.39.1,^22^), denoising (IPED, v.1.0,^23^), chimera-removal (CATCh, v.1.0,^24^) and operational taxonomic units (OTUs) clustering using UPARSE with 97% clustering cut-off.^25^ The alignment was performed against the SILVA database (v.119,^26^) and OTUs were classified using the Ribosomal Database Project (RDP) dataset (v.16,^27^) with 80% cut-off. Then, samples were rarified to a depth of 14999 reads as in the smallest sample depth. Alpha diversity indices were calculated using Shannon, Chao and Shannon even. Beta diversity was estimated through UniFrac unweighted analysis using the mothur *dist.shared* command. These distances were visualized using non-metric multi–dimensional scaling (NMDS), using the mothur *nmds* command. Statistical comparison of alpha diversity was conducted using linear mixed effects models to account for repeated measures, whereas Analysis of Molecular Variance (AMOVA; i.e., ANOVA-like statistical method developed for metagenomic datasets) was used to compare beta diversity between samples using the mothur *amova* command. Further analyses and visualization were performed using the *Rhea* package (v.1.6,^28^) and other in-house scripts (using *ggplot2* package) in RStudio (v.3.5.0).

### Taxonomic discovery analysis

Differentially abundant OTUs of the irradiated microbial community were determined by a random intercept model as implemented in the analysis of composition of microbiomes (ANCOM^29^) Based on the additive log ratio transformed OTU frequencies, a *W*-statistic was calculated when comparing post-irradiation samples (PID1, PID3 and PID7) to the initial microbial community at PID0. Finally, OTUs with a ln(fold change) ≥1 and a *W*-statistic ≥0.7 were considered as differentially abundant and depicted as markers of dysbiosis.

The purity of these OTUs were assessed using oligotyping approach,^30^ which can distinguish up to a single nucleotide difference in the reads compromising each of those OTUs. The identified oligotypes were classified with NCBI Nucleotide BLAST using default settings and the RDP database as reference.^31^

### Statistical analyses

Data were processed, analyzed and visualized (in boxplots) using R studio software packages including *ggplot2* and *ggsci*. Outliers as defined by the Tukey’s fences criteria (i.e., values below Q1 − 1.5*IQR or above Q3 + 1.5*IQR) were excluded from further statistical analyses. Statistical significance was determined using (generalized) linear models. Repeated measures data analysis was performed using (generalized) linear mixed models. Based on the model outcomes, obtained ß coefficients represented the change in the outcome variable for every unit of change in the predictor variable assuming other variables were held constant, and were accompanied with a *p* value. Differences with *p* < 0.05 were considered statistically significant.

## Results

### Pelvic irradiation affects body weight and induces morphological changes in the intestine

**Fig. 1:**
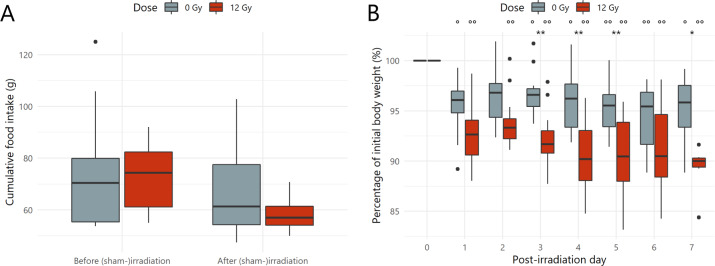
Pelvic irradiation affects body weight. **A** Comparison of cumulative food intake seven days before and after (sham-)irradiation, *n* = 14 per group. Non-significant *p* values by linear modeling. **B** Percentage of initial body weight over time after (sham-)irradiation, *n* ≥ 7 per group. *p < 0.05, **p < 0.01 for time independent differences by linear modeling and °*p* < 0.05, °°*p* < 0.01 for time dependent differences relative to the day of (sham-)irradiation (Post-irradiation day 0) by linear mixed effects modeling.

Firstly, cumulative food intake over the 14-day experimental period was not shown to be affected by the (sham-)irradiation procedure (Fig. 1A). Next, body weight relative to the initial weight at the day of (sham-)irradiation (PID0) was investigated, revealing a significantly decreased body weight in both sham-irradiated and irradiated mice, which was exacerbated upon irradiation at PID3, 4, 5 and 7 (Fig. 1B).

**Fig. 2:**
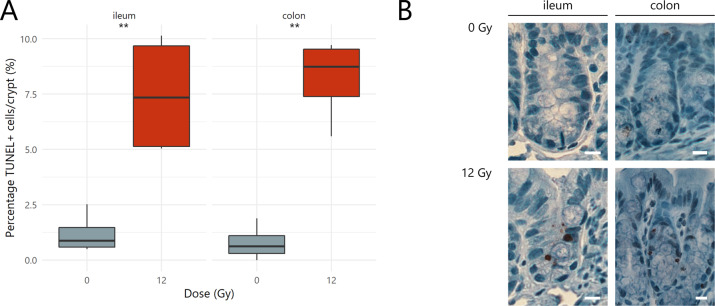
Pelvic irradiation rapidly induces apoptosis in the intestine. **A** Crypt apoptosis index representing the percentage of TUNEL+ cells per total number of crypt cells for ileum and colon one day following (sham-)irradiation, *n* = 4 per group. **B** Representative images of TUNEL staining. Brown nuclei are TUNEL+ cells and scale bars represent 10 µm. **p < 0.01 by linear modeling.

At PID1, histopathology revealed a significant increase in the percentage of apoptotic nuclei in all intestinal crypts of 12 Gy-exposed mice as compared to sham-irradiated mice (Fig. 2A, B).

**Fig. 3:**
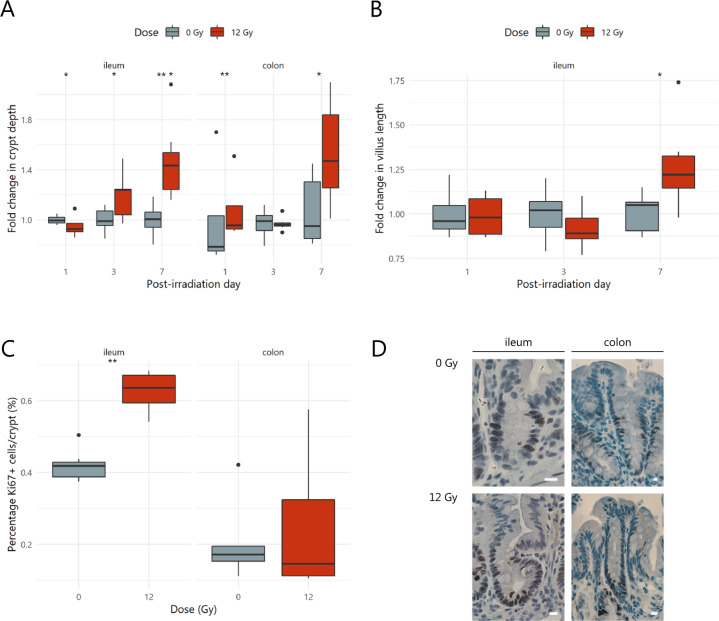
Pelvic irradiation induces morphological changes in the intestine. Boxplots showing the fold changes in mucosal parameters including (**A**) crypt depth and (**B**) villus length following pelvic (sham-)irradiation, *n* = 4–7 per group. **C** Crypt proliferation index representing the percentage of Ki67+ cells per total number of crypt cells for ileum and colon seven days following (sham-)irradiation, *n* = 7 per group. **D** Representative images of Ki67 staining. Brown nuclei are Ki67+ cells and scale bars represent 10 µm. *p < 0.05, **p < 0.01 by linear modeling.

Since the proliferative crypt environment was affected by apoptosis, the effects of pelvic irradiation on the mucosal barrier were investigated. For instance, villus length and crypt depth are well-described intestinal parameters damaged by radiation exposure. When compared to sham-irradiated samples, ileal tissue was strongly affected by irradiation through a temporarily decreased crypt depth at PID1, which then increased from PID3 onwards (Fig. 3A). At PID7, this increased crypt depth was corroborated by lengthened ileal villi (Fig. 3B). In colonic tissues, irradiation-induced enlargement of crypts was observed at PID1 and PID7 (Fig. 3A). These mucosal changes are indicative of repair following injury, which is likely associated with an overshoot in cell production.^18^ Such a compensatory rise in cell proliferation was indeed confirmed at PID7 in the ileum, but not in the colon, as shown by an increased number of Ki67-positive nuclei (Fig. 3C, D). These data support the significant regeneration properties of the intestine activated after a physicochemical insult such as pelvic irradiation.

### Pelvic irradiation evokes a rapid inflammatory response

**Fig. 4:**
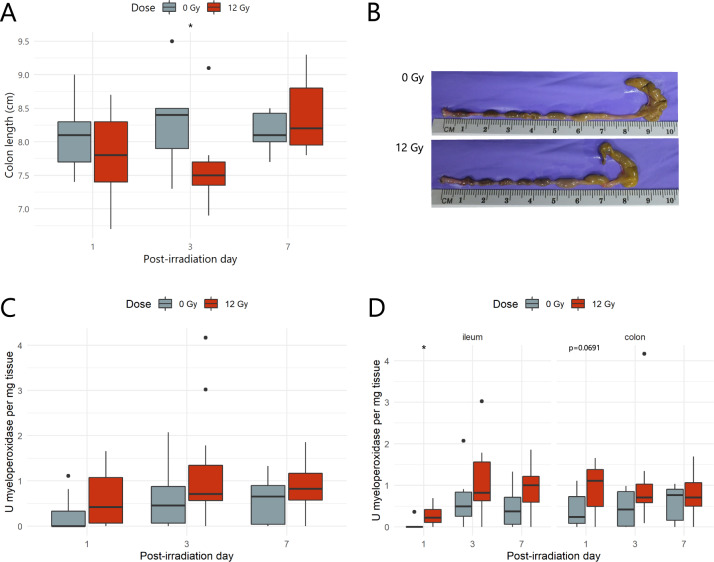
Pelvic irradiation evokes a rapid inflammatory response. **A** Boxplots showing the quantitative colon length measurements, *n* = 6–7 per group. **B** Representative images of murine colon of sham- and irradiated mice. Inflammatory myeloperoxidase activity investigated (**C**) irrespective of the intestinal segment, and (**D**) in ileum and colon separately after (sham-)irradiation, *n* = 6–7 per group. *p < 0.05 by linear modeling.

Next, pelvic irradiation was investigated for its effects on macroscopic and molecular inflammatory parameters. First, macroscopic analysis revealed a significant shortening of the colon length at PID3 (Fig. 4A, B), a hallmark of rodent intestine inflammation.^32^ Furthermore, molecular analyses showed a main effect of radiation exposure (ß = 0.03; *p* = 0.008) on intestinal myeloperoxidase activity (Fig. 4C). Yet, different intestinal regions were shown to have a distinct susceptibility to develop an inflammatory response following radiation exposure. Specifically, ileal myeloperoxidase activity was transiently affected at PID1, whereas, despite macroscopic signs of inflammation, myeloperoxidase activity was not altered upon irradiation in the colon (Fig. 4D).

### Impaired barrier integrity following pelvic irradiation enhances bacteria to translocate to mesenteric lymph nodes

**Fig. 5:**
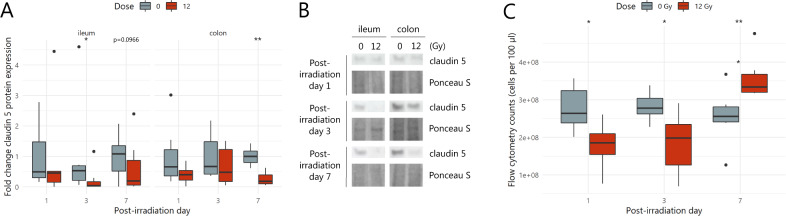
Pelvic irradiation functionally impairs the intestinal barrier. **A** Effects of pelvic irradiation on claudin 5 (23 kDa) tight junction protein expression in ileum and colon, *n* = 6–7 per group. **B** Representative western blot images of claudin 5 and Ponceau S total protein staining, *n* = 6–7 per group. **C** Bacterial counts obtained by flow cytometry in mesenteric lymph nodes after (sham-)irradiation, *n* = 6–7 per group. *p < 0.05, **p < 0.01 by linear modeling.

In an attempt to correlate the irradiation-induced morphological and inflammatory changes to overall intestinal barrier functionality, its integrity and permeability were assessed by evaluating tight junction expression and bacterial translocation (i.e., from intestinal lumen into mesenteric lymph nodes), respectively. First, the expression of claudin 5, a transmembrane protein contributing to barrier sealing properties of epithelial tight junctions,^33^ was investigated by western blot. In the ileum, claudin 5 expression was significantly decreased at PID3, whereas its colonic expression was lowered only at PID7 (Fig. 5A, B). To assess its consequence on barrier permeability, bacterial translocation into mesenteric lymph nodes was evaluated. Over all time points, an equivalent bacterial load was counted in mesenteric lymph nodes of sham-irradiated mice, which was considered as the baseline bacterial translocation in healthy mice (Fig. 5C). Of interest, mesenteric lymph nodes’ bacterial counts decreased promptly following 12 Gy exposure. This was followed by a shift in absolute counts at PID7, showing a higher translocation rate of luminal bacteria into mesenteric lymph nodes in irradiated mice compared to sham-irradiated mice (Fig. 5C). These data thus suggest that pelvic irradiation-induced structural changes in tight junction proteins accounting for a functionally disturbed intestinal barrier.

### 16S rRNA gene sequencing coverage

In the microbiome 16S rRNA sequencing data set, the alpha rarefaction curve showed that gene richness approached overall saturation as a function of number of reads, indicating that the number of reads was sufficient to capture the vast majority of the OTUs present (Supplementary Fig. 2A).^34^ To support this and ensure good estimation of bacterial diversity, we measured the proportion of OTUs represented in samples of each group by the Good’s estimator of coverage for an OTU definition of 0.03.^25,34^ The average of estimated coverages was 99.93 ± 0.03% for all samples, suggesting that the 16S rRNA results from each library (i.e., sham- and irradiated fecal microbiome at PID0, PID1, PID3 and PID7) represented an adequate level of sequencing to identify most diversity in the samples (Supplementary Fig. 2B). Following rarefaction, the obtained reads were linked with a total of 391 OTUs, and 126 ± 19 OTUs on average per sample.

### The intestinal bacterial microbiome profile changes following pelvic irradiation

**Fig. 6:**
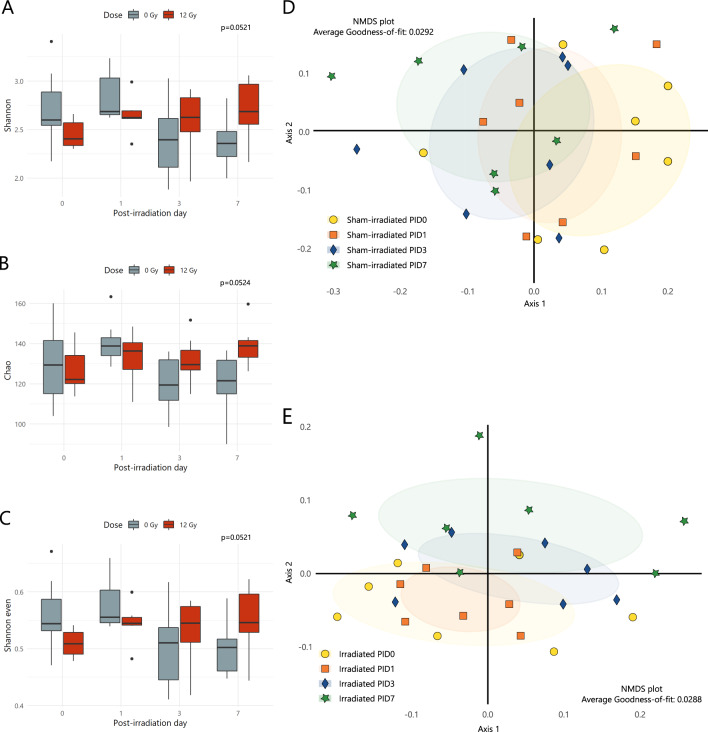
Pelvic irradiation impacts diversity of gut microbiome in a delayed fashion. Microbial alpha diversity index representing the diversity within a fecal sample, considering (**A**) both richness and evenness (Shannon), (**B**) solely richness (Chao) or (**C**) solely evenness (Shannon even) of sham- and irradiated mice, *n* = 7 per group. Non-significant *p* values by linear modeling. NMDS plot of unweighted UniFrac beta analysis of (**D**) sham- and (**E**) irradiated mice showing the diversity between samples without consideration of relative abundances, *n* = 7 per group. PID = post-irradiation day.

To evaluate potential alterations in the microbial communities between sham- and irradiated mice, microbial alpha diversity (i.e., diversity within a sample, considering both richness and evenness) and beta diversity indices (i.e., diversity between samples without the consideration of abundances) were calculated. No effects on alpha diversity metrics were observed in irradiated mice when compared to sham-irradiated mice (Shannon, Chao and Shannon even; Fig. 6A–C). Microbiome communities were then further compared by a distance matrix based on unweighted UniFrac beta diversity index with 1000 permutations (considering microbial membership). When comparing microbiome baselines of sham-irradiated and irradiated mice at PID0, differences were observed despite the major efforts taken to reduce the effects of confounding factors (see ‘Mice’ methods section) (Supplementary Fig. 3). Therefore, further analyses were performed in a paired manner, thus focusing on the sham- and irradiated cohorts over time and capturing the individual variation for each mouse over time. Analyses of the sham-irradiated microbial community representing the effects induced by anesthesia indicated no significant shift of beta diversity (PID0 vs PID1 *p* = 0.394; PID0 vs PID3 *p* = 0.376; PID0 vs PID7 *p* = 0.058 by AMOVA) compared to the initial microbial community at PID0 (Fig. 6D). In contrast, the NMDS plot of unweighted UniFrac index showed that samples of irradiated mice collected at PID3 and PID7 clustered differently compared to the PID0 and PID1 samples, being the most prominent at PID7 (Fig. 6E). The latter differences were supported by AMOVA based on unweighted UniFrac distance (PID0 vs PID1 *p* = 0.858; PID0 vs PID3 *p* = 0.025; PID0 vs PID7 *p* = 0.004).

### Bacterial markers of dysbiosis are identified following pelvic irradiation

Next, we compared the microbial composition at various taxonomic levels between mice before (at PID0) and after pelvic irradiation (at PID1, PID3 and PID7) (Supplementary Fig. 4A–E). To identify the specific OTUs significantly associated with pelvic irradiation, the composition of fecal microbiota was investigated using ANCOM. This tool generated a list of 25 OTUs, belonging to the *Ruminococcaceae*, *Lachnospiraceae* and *Porphyromonadaceae* families (Table 1). In a next instance, the OTUs predefined as dysbiosis markers were further refined using oligotyping. For OTU102, two distinct oligotypes could be identified, which resulted in a different taxonomic classification using NCBI blast, as shown by a percentage identity of ~97% and 100% coverage (Table 1). For the remainder of the OTUs, it was not possible to stratify them further into oligotypes with distinct taxonomic classifications indicating that each of these OTUs constitutes a single taxonomic unit.

**Table 1:** Significant gut microbial dysbiosis markers detected in mice following pelvic irradiation.

Taxonomic classification (following Ribosomal Database Project)	ANCOM biomarkers’ effect size and *W*-statistic	Highest NCBI Blast hit (% identity)^a^
*Ruminococcaceae*_OTU9	1.58 – *W* = 0.9 (PID1); 1.70 – *W* = 0.9 (PID3); 1.40 – *W* = 0.9 (PID7)	
*Bacteroides*_OTU22	1.22 – *W* = 0.7 (PID1)	*Bacteroides acidifaciens* (>99%)
*Lachnospiraceae*_OTU31	1.21 – *W* = 0.9 (PID7)	
*Oscillibacter*_OTU135	1.39 – *W* = 0.9 (PID3)	
*Anaerotruncus*_OTU202	1.18 – *W* = 0.9 (PID3)	
*Butyricicoccus*_OTU220	1.12 – *W* = 0.9 (PID1)	
*Clostridium*_XlVb_OTU225	1.11 – *W* = 0.9 (PID3); 1.11 – *W* = 0.9 (PID7)	*Anaerotignum lactatifermentans* (>97%)
*Ruminococcaceae*_OTU258	1.19 – *W* = 0.9 (PID3)	
*Lachnospiraceae*_OTU298	1.23 – *W* = 0.9 (PID7)	
*Clostridiales*_OTU102	−1.12 – *W* = 0.9 (PID1)	*Flintibacter butyricus* (~97%)
		*Pseudoflavonifractor capillosus* (~97%)
*Lachnospiraceae*_OTU148	−1.36 – *W* = 0.9 (PID3)	
*Desulfovibrionaceae*_OTU149	−1.01 – *W* = 0.9 (PID7)	
*Porphyromonadaceae*_OTU181	−1.35 – *W* = 0.9 (PID1); −1.47 – *W* = 0.9 (PID3); −1.01 – *W* = 0.9 (PID7)	
*Clostridium*_IV_OTU186	−1.49 – *W* = 0.9 (PID7)	
*Lachnospiraceae*_OTU215	−1.20 – *W* = 0.9 (PID1)	
*Clostridiales*_OTU233	−1.37 – *W* = 0.9 (PID3); −1.14 – *W* = 0.9 (PID7)	
*Clostridiales*_OTU239	−1.14 – *W* = 0.9 (PID7)	
*Porphyromonadaceae*_OTU240	−1.21 – *W* = 0.9 (PID7)	
*Lachnospiraceae*_OTU242	−1.17 – *W* = 0.9 (PID3)	
*Bacteria*_OTU251	−1.46 – *W* = 0.9 (PID3)	
*Firmicutes*_OTU257	−1.21 – *W* = 0.9 (PID3)	
*Porphyromonadaceae*_OTU261	−1.30 – *W* = 0.9 (PID1)	
*Ruminococcaceae*_OTU267	−1.50 – *W* = 0.9 (PID1); −1.19 – *W* = 0.9 (PID3)	
*Clostridiales*_OTU274	−1.00 – *W* = 0.9 (PID7)	
*Lachnospiraceae*_OTU286	−1.12 – *W* = 0.9 (PID7)	

PID = post-irradiation day.

^a^If >1 oligotype was detected, highest NCBI Blast hits are shown for all oligotypes.

## Discussion

In this work, we sought to provide insights in the interplay between the host and its microbiome following pelvic radiation exposure. In particular, we attempted to characterize pelvic irradiation-induced intestinal mucositis, a common clinical side effect of pelvic radiotherapy, along with a thorough investigation of the microbiome response.

**Fig. 7:**
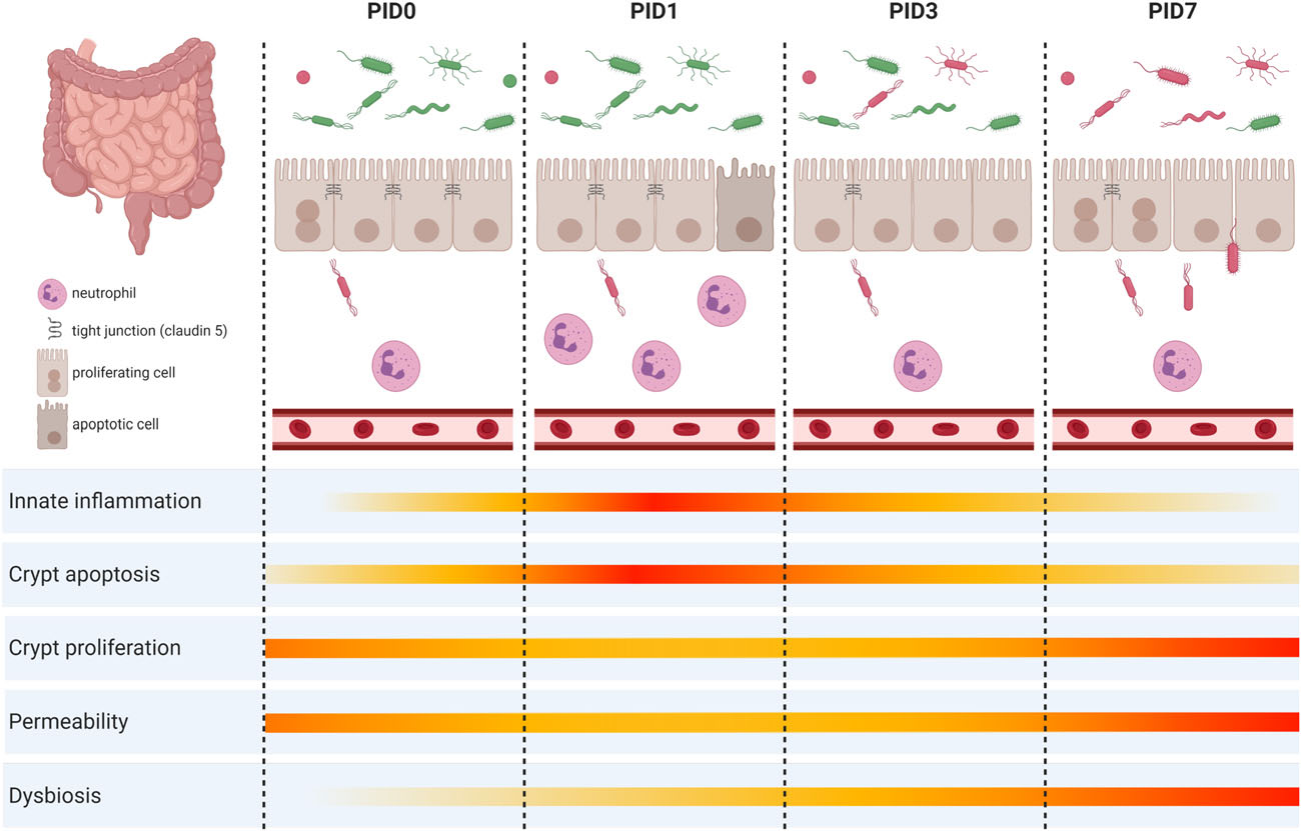
Graphical summary of the main results illustrating the impact of pelvic irradiation on the healthy intestine and the residing microbiome based on the different parameters monitored in this study. Pelvic irradiation showed morphological and inflammatory implications, functionally impairing the intestinal barrier. Concurrent 16S microbial profiling revealed a delayed impact of pelvic irradiation on the composition of the intestinal microbial community excluding a causal role of dysbiosis in the initiation of intestinal mucositis. This figure was created with BioRender.com. PID = post-irradiation day.

The pathobiology of mucositis following pelvic irradiation described here, perfectly followed the sequence of biological events as described by Cinausero et al.^35^ Briefly, pelvic irradiation initiated a primary damage response in ileum and, to a lesser extent, in colon including apoptosis and inflammation as shown by histology and myeloperoxidase activity, respectively. These destructing signals then amplified leading to discontinuity of the epithelial barrier, characterized by loss of tight junctions, promoting bacterial translocation into mesenteric lymph nodes. Eventually, healing processes were activated through enhanced Ki67-mediated cell proliferation once irradiation has ceased. Furthermore, unprecedented findings showed that dysbiosis developed secondarily to these structural and functional changes in the irradiated intestine. For an overview of this sequence of events, we refer to Fig. 7.

Mild post-anesthetic weight loss, as observed in our study following sham-irradiation, is commonly reported and might persist for several days likely because of depressed food and water intake.^36,37^ An even greater weight loss was induced by pelvic irradiation, as reported earlier.^17,38^ Irradiation-induced cytotoxic effects are related to various DNA damaging actions. Despite an immediate increase in intestinal crypt apoptosis and short-term depletion of ileal crypts, subsequent shortening of villi was not observed in our study. This is in contrast to previous reports and is likely due to the dramatic effects evoked by the irrational use of total body irradiation.^39,40^ Also, colonic morphology appeared less affected by pelvic irradiation, likely due to higher radioresistance as previously reported.^41^ Seven days following pelvic irradiation, our study confirmed the activation of crypt regeneration in ileum and colon by histology. This suggests that our procedure also allowed for a sufficient amount of surviving crypts to restore intestinal morphology.^17,42^ However, an increased crypt proliferation rate, to support the deepened crypts at PID7, was not observed in the likely more radioresistant colonic epithelial cells, as reported earlier.^41^ Besides irradiation-induced apoptosis, an acute inflammatory response, shown by ileal myeloperoxidase activity indicative of mucosal neutrophil infiltration, and a significant shortening of the colon length was demonstrated in our study. This rapid induction of inflammation is in line with previous radiation exposure studies.^39,43,44^ In all, these data provide evidence for a primary damage response in ileum and, to a lesser extent, in colon, in terms of apoptosis and inflammation evoked by pelvic irradiation.

As previously stated, the combination of apoptosis and inflammation early after pelvic irradiation is expected to impair the intestinal barrier enhancing bacterial translocation from the intestinal lumen into surrounding mesenteric lymph nodes.^45^ Here, evidence for irradiation-induced barrier impairment and bacterial translocation was shown by tight junction expression analysis, as described previously,^39,46,47^ and the innovative use of flow cytometry. Initially, when compared to sham-irradiated mice, a decreased bacterial translocation was observed likely due to the antimicrobial actions of neutrophils, crucial in the first control action of intestinal inflammation.^44^ Irradiation-induced short-term reduction of the bacterial load in the small intestine was also observed by Brook et al.^48^ However, excessive and sustained reactive oxygen species production likely aggravates intestinal damage, disrupting tight junctions as a part of the intercellular barrier. This increases the opportunity for surviving bacteria to translocate to mesenteric lymph nodes, as we observed seven days following pelvic irradiation, and as reported using plate counting.^43,49^ These data highlight the effect of pelvic irradiation on intestinal barrier integrity and bacterial translocation in vivo, which may entail the dissemination of pathogens throughout the body as well as of the development of chronic effects.

Having shown evidence for the initiation of intestinal mucositis within one day following pelvic irradiation, we reported delayed effects on the composition of gut microbiota. Although low bacterial richness has often been associated with intestinal exposure to radiation,^50–52^ we could not report an equivalent reduction in alpha diversity metrics. Therefore, we could hypothesize that a reduction in alpha diversity or richness is not expected following irradiation, which was also observed in mice and patients developing irradiation-induced mucositis.^2,15,53^ Yet, supporting our hypothesis of an irradiation-induced shift in microbial beta diversity, a shift in unweighted UniFrac beta diversity was observed, as previously described.^12,15,53^

Accounting for compositionality, the unraveling of relevant OTUs uniquely affected by pelvic irradiation in a delayed fashion highlighted members of the *Ruminococcaceae* family, which were also reported to increase in mice following repeated irradiations,^52^ and were even described to be radiation-resistant, together with *Lachnospiraceae* and *Clostridiaceae*, following sub-lethal murine exposure.^54^ Different responses following irradiation were observed for members belonging to the *Lachnospiraceae* family, which corresponds with reported cases of intestinal damages.^55–57^ Within both *Ruminococcaceae* and *Lachnospiraceae* families, significant increases were observed in OTUs associated with genera *Anaerotruncus* as well as *Oscillibacter* and *Clostridium* cluster XIVb, being in line with what is reported in irradiated mice and minipigs, respectively.^12,52,57,58^ Of interest, the authors reported a positive correlation between *Oscillibacter* abundance and radiation intensity in larger animals.^58^ Higher abundances of *Oscillibacter* spp. and *Anaerotruncus* spp. were also noted in mice and humans with inflamed and hyper-permeable intestines.^59–64^ In contrast, the relative abundances of OTUs belonging to the *Porphyromonadaceae* family were significantly decreased in our study, as previously reported in repeatedly irradiated mice and mice with inflammatory bowel disease.^52,65,66^ These identified biomarkers may be used for disease diagnosis and/or prognosis, as well as personalized treatment development in cases of irradiation-induced intestinal complications.

Important in our study, the unique inclusion of an early time point (PID1) following single exposure of the pelvis could show no active role for the intestinal microbiome in the initiation of intestinal mucositis since no changes in microbial communities were yet detected (Fig. 7). This noted paradigm shift is particularly important for future metagenomic predictions of certain diseases as associations may not imply causality. Nevertheless, the occurrence of bacterial translocation following pelvic irradiation due to loss of epithelial integrity, as observed in our study, may provide an opportunity for the dysbiotic community to evoke chronic intestinal responses, as postulated earlier.^3^ In line with this, previous studies demonstrated the central role of the epithelial barrier in bacterial sensing by expressing pattern recognition receptors such as toll like receptors, which can trigger inflammatory responses upon its interaction with gut microbiota.^67^ Future metatranscriptomic and metabolomic investigations will allow for further validation of both acute and chronic radiation pathways, and identification of clinically relevant biomarkers.

In conclusion, by applying a multi-level approach we documented a rapidly induced crypt epithelial cell death and inflammatory response in ileum and colon following acute pelvic irradiation, concomitant with a disturbed barrier integrity and translocation of intraluminal bacteria into mesenteric lymph nodes as depicted by the innovative use of flow cytometry (Fig. 7). A secondary effect following irradiation-induced intestinal mucositis involved a delayed, yet significant, impact on gut microbial beta diversity characterized by dysbiosis markers unique for pelvic irradiation including members of the *Ruminococacceae*, *Lachnospiraceae* and *Porphyromonadaceae* families. This in vivo irradiation-gut-microbiome test platform may stimulate fundamental research as well as development of treatments (i.e., radioprotective drugs and food supplements) to support patients’ quality of life following radiotherapy.

## Supplementary Information

Supplementary materials and method
