# Supplementary material for: Intestinal mucositis precedes dysbiosis in a mouse model for pelvic irradiation
Source: ISME Commun. 2021 Jun 10;1:24. doi: 10.1038/s43705-021-00024-0 (PMC9723693; doi:10.1038/s43705-021-00024-0)
Supplement: Supplementary file 1 — Supplementary materials and method [file 43705_2021_24_MOESM1_ESM.docx]

### Supplementary material and methods

### Histology and immunohistochemistry

Distal ileum and proximal colon were rinsed with ice cold phosphate buffered saline (GibcoTM, Thermo Fisher Scientific, Belgium) and fixed in 4% paraformaldehyde (Merck, Belgium). Histology and immunohistochemistry was performed on 5 µm paraffin-embedded tissues cut on a Thermo Scientific HM 340E Electronic Rotary Microtome perpendicular to the long axis of the intestine and mounted onto SuperfrostTM microscope slides (Thermo Fisher Scientific, Belgium).

General morphology of intestinal samples was assessed by hematoxylin and eosin staining. Slides were imaged using a bright‐field Nikon Ti‐Eclipse microscope and a 20x objective. Villus length and crypt depth were quantified at 10 random places within 10 intestinal tissue sections per mouse (blindly coded).

Immunohistochemical identification of the nuclear protein Ki67, a marker for cell proliferation, was performed using polyclonal rabbit anti-mouse Ki67 antibody (#Ab15580, Abcam, UK), following standard protocols supplied by the manufacturer. Briefly, sections were de-paraffinized and rehydrated. Microwave mediated antigen retrieval was performed in DAKO Retrieval Solution (#S1699, Agilent, Belgium). After quenching endogenous peroxidase with methanol and 0.3% hydrogen peroxidase as well as blocking nonspecific bindings with goat pre-immune serum (Thermo Fisher Scientific, USA), sections were incubated overnight with primary antibody diluted (1:200) in Tris-NaCl-blocking buffer. Tissues were then incubated with secondary HRP-linked goat anti-rabbit antibody for 2 hours.

Ionizing radiation is known to induce apoptosis of intestinal crypt epithelial cells [1]. Therefore, histopathology was performed at PID1 on ileal and colonic crypt epithelial cells using Terminal deoxynucleotidyl transferase-mediated dUTP nick end labeling (TUNEL). The In Situ Cell Death Detection Kit (#11684817910 Roche, Merck, Belgium) was used according to manufacturer’s instructions.

For visualization, the DAKO Envision+ HRP (DAB, Thermo Fisher Scientific, Belgium) system was used. Slides were imaged using a bright-field Nikon Ti‐Eclipse microscope and a 20x objective. The number of positively stained cells were counted in 50 random crypts per mouse (blindly coded) and normalized to the total number of cells in the crypts.

### Intestinal myeloperoxidase activity assay

To monitor the degree of acute inflammation, myeloperoxidase activity was measured in intestinal tissues. Specifically, myeloperoxidase activity is related to the number of neutrophil infiltrates [2]. Ileal and colonic myeloperoxidase activity was measured as described by Breugelmans T. *et al*. (2020) [3]. Briefly, intestinal segments were blotted dry, weighed, and placed in hexadecyltrimethylammonium bromide (HTAB) buffer (0.5% HTAB (Sigma-Aldrich, Belgium) in 50 mM phosphate buffer, pH 6.0) at a ratio of 5 g of tissue per 100 mL of buffer. The samples were maintained on ice and homogenized twice for 30 seconds at 25 Hz/s using a Tissue Lyser (Qiagen, The Netherlands). The homogenates were then centrifuged at 15 000 xg for 15 minutes at 4°C. The supernatants were assayed for myeloperoxidase activity by adding aliquots of 10 µL to 290 µL of O-dianisidine solution (0.167 mg/mL O-dianisidine hydrochloride (Sigma-Aldrich, Belgium) and 0.0005% hydrogen peroxide in 50 mM phosphate buffer, pH 6.0). The change in absorbance was read at 460 nm over 60 seconds with a CLARIOstar Plus microplate reader (BMG Labtech, Isogen LifeScience, The Netherlands). Data represent units per gram of tissue in which one unit equals the amount of myeloperoxidase necessary to degrade 1 µmol of hydrogen peroxide per minute at 25°C.

### Western blot analysis of claudin 5

Total cell lysates from intestinal samples were prepared in ice-cold RIPA buffer (50 mM Tris-HCl (pH 7.4), 150 mM NaCl, 1% Triton X-100, 0.5% sodium deoxycholate, 0.1% sodium dodecyl sulfate, 1mM protease inhibitor, 1mM phosphatase inhibitor). Protein concentrations were determined by bicinchoninic acid assay (Sigma-Aldrich, Belgium). Cell extracts were subjected to sodium dodecyl sulfate polyacrylamide gel electrophoresis. Here after, proteins were transferred to nitrocellulose membranes and probed with primary antibodies detecting the tight junction protein claudin 5 (1:500; 23 kDa, #35-2500, Invitrogen, USA) and with secondary HRP-linked goat anti-mouse antibodies (#P0447, DAKO, Agilent, Belgium). Signals were detected with enhanced chemiluminescence (Biorad, Belgium). Reversible Ponceau S stain (Sigma-Aldrich, Belgium) was used, according to manufacturer’s instructions, to confirm equal protein loading and normalize protein abundance. The blots were imaged using a Fusion FX imager (Vilber Lourmat, France). Intensities of protein bands were semi-quantified using ImageJ software.

### Supplementary figures


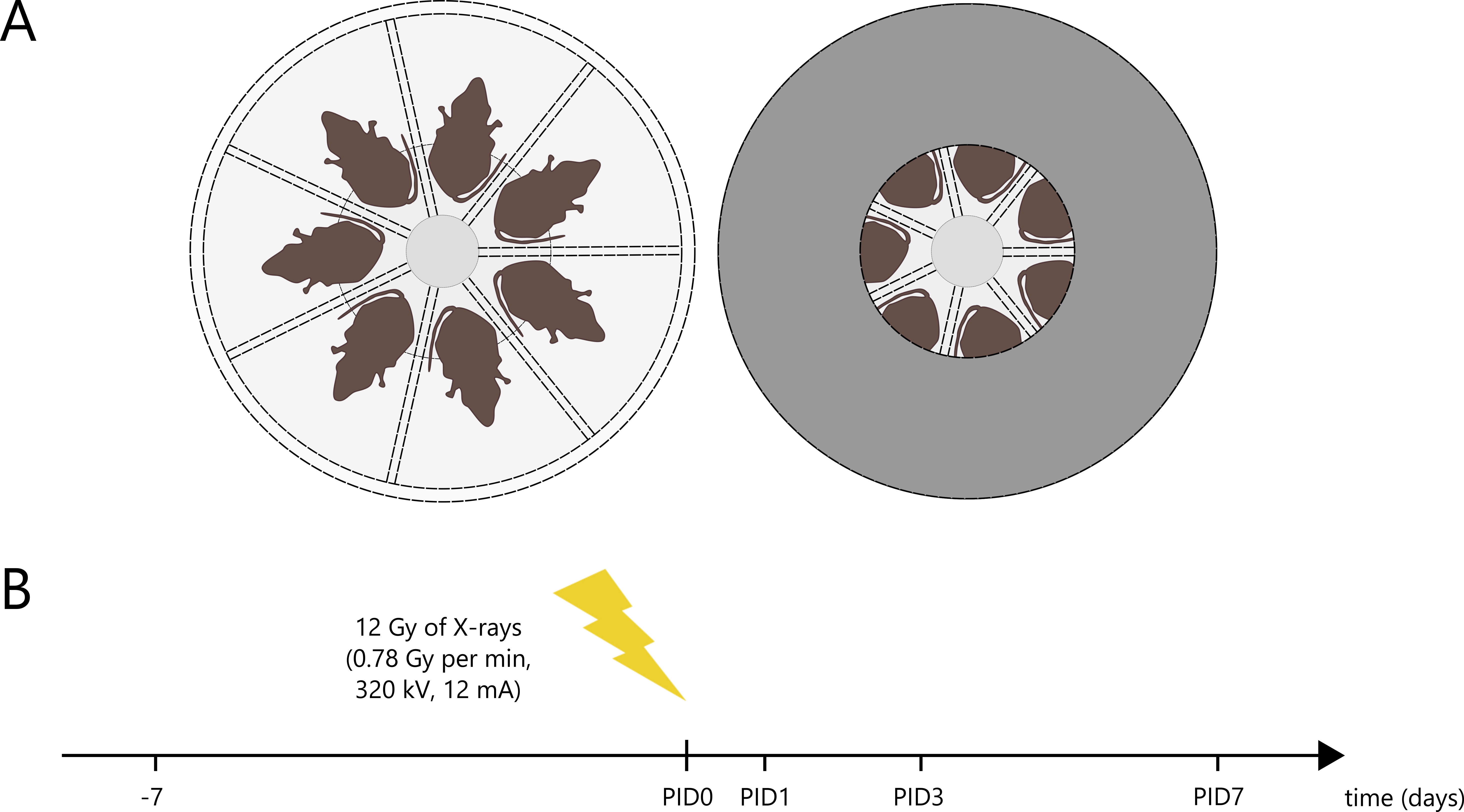


Supplementary figure 1. A,B) Experimental setup for pelvic irradiation of mice. A) Disk-shaped Plexiglas box used for local, pelvic irradiation of the mice. Individual animals were placed in a prone position with their lower body parts towards the center of this box (left picture). The entire box was covered by a lead shield (5 mm thick) except for the center of the cover (9 cm diameter) (right picture). B) Experimental time line for pelvic irradiation of mice. PID=post-irradiation day


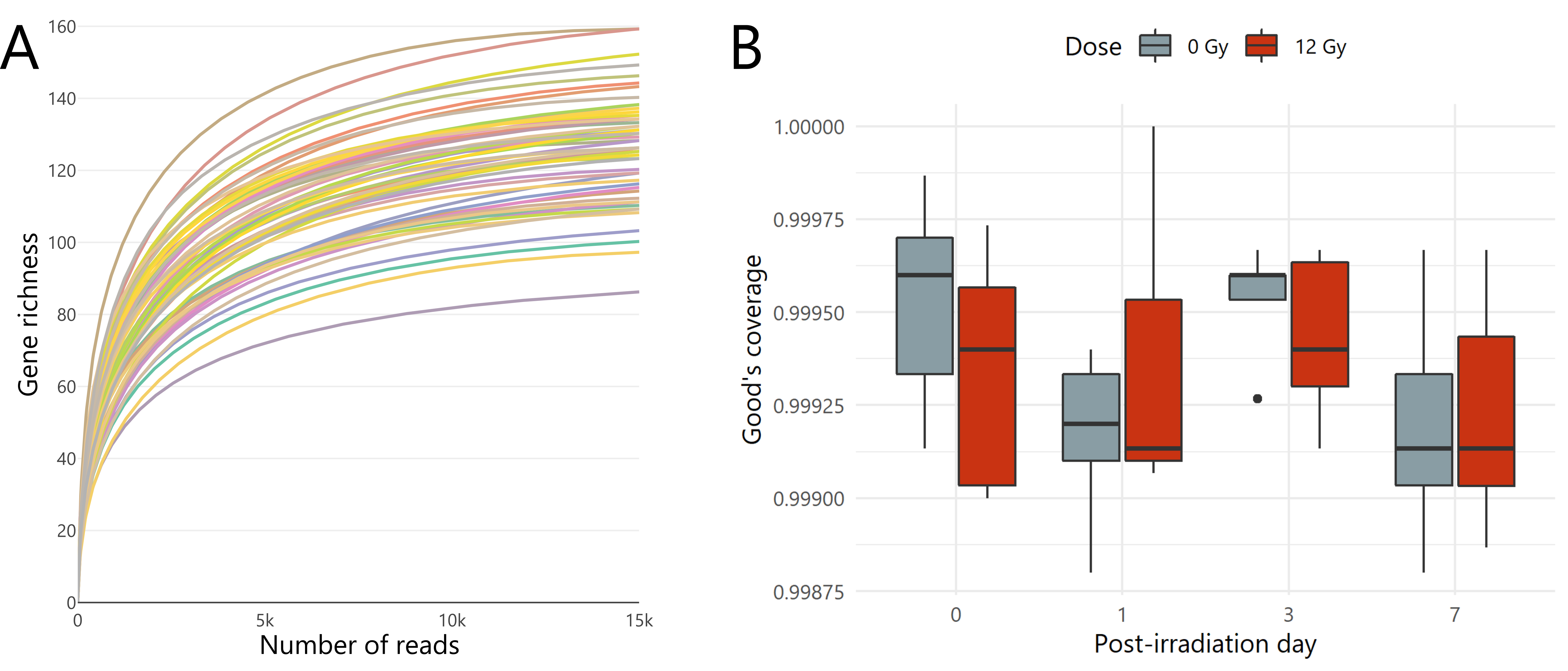


Supplementary figure 2. A,B) An adequate depth of sequencing was reached to identify most diversity in the samples. A) Rarefaction curve displaying gene richness as a function of the number of reads per sample. B) Good’s estimator of coverage as a measure of sample completeness.


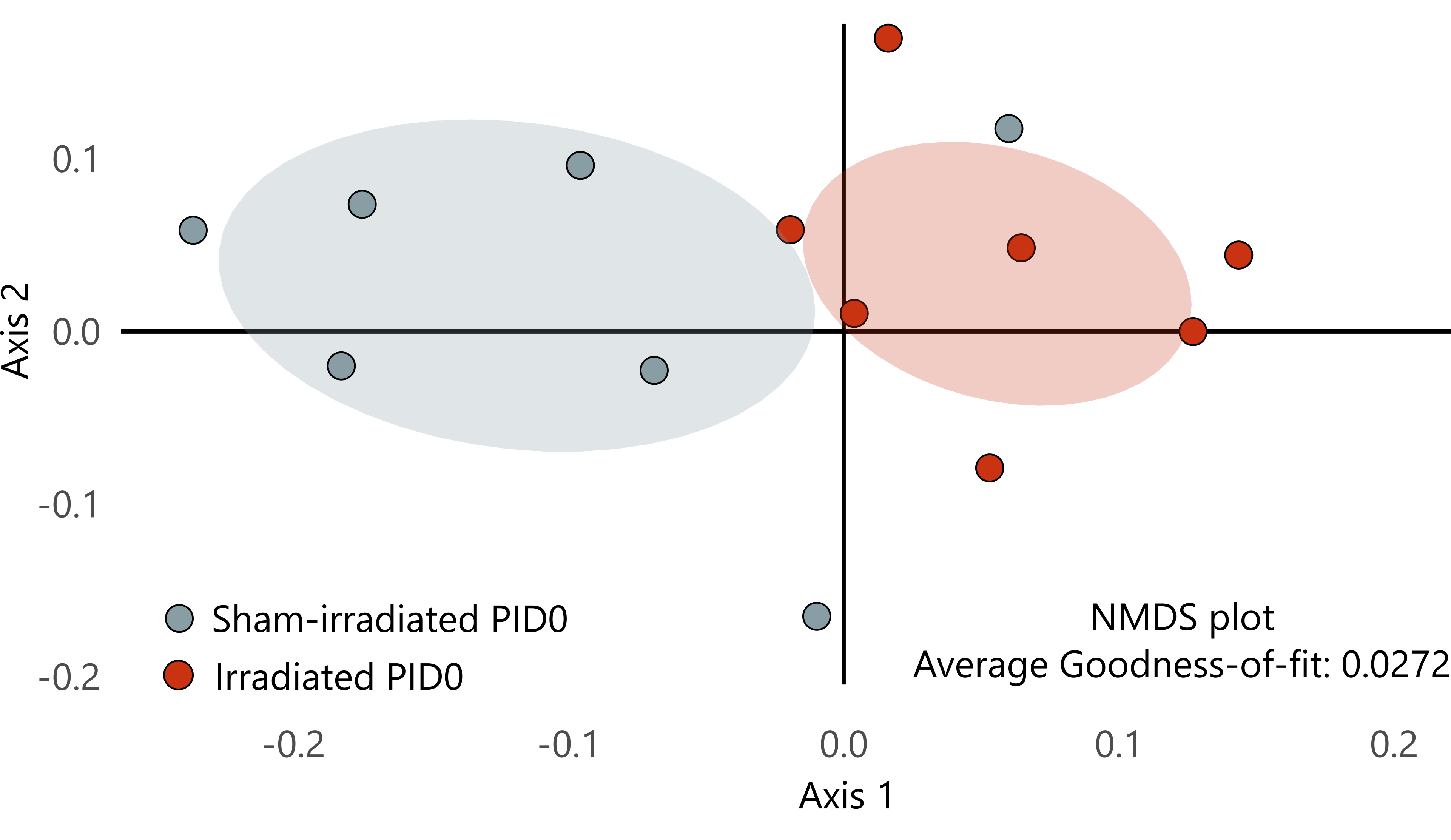


Supplementary figure 3. Unweighted UniFrac NMDS plots displaying baseline inter-sample diversity at post-irradiation day 0 among sham- and irradiated cohorts. PID=post-irradiation day


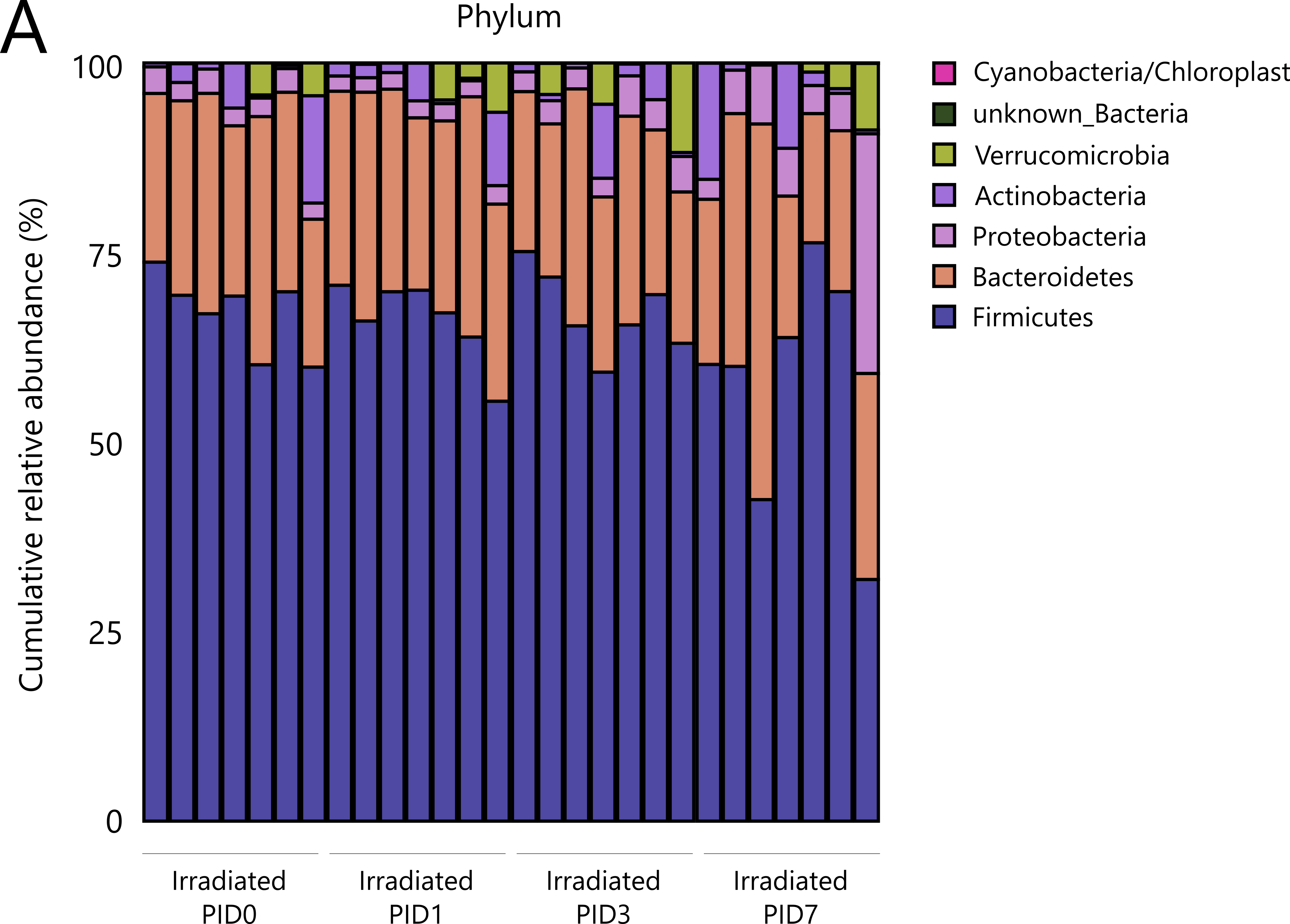


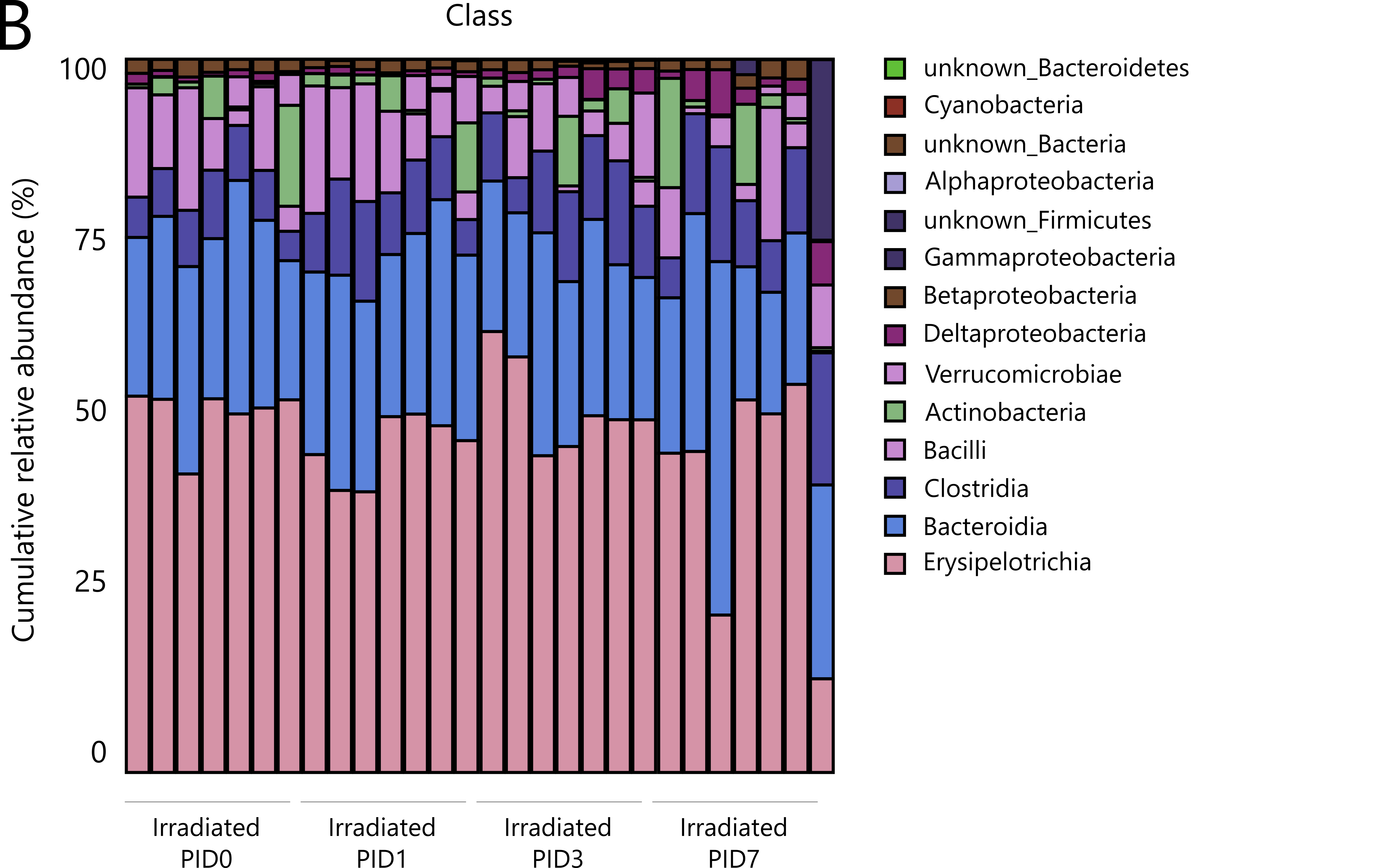


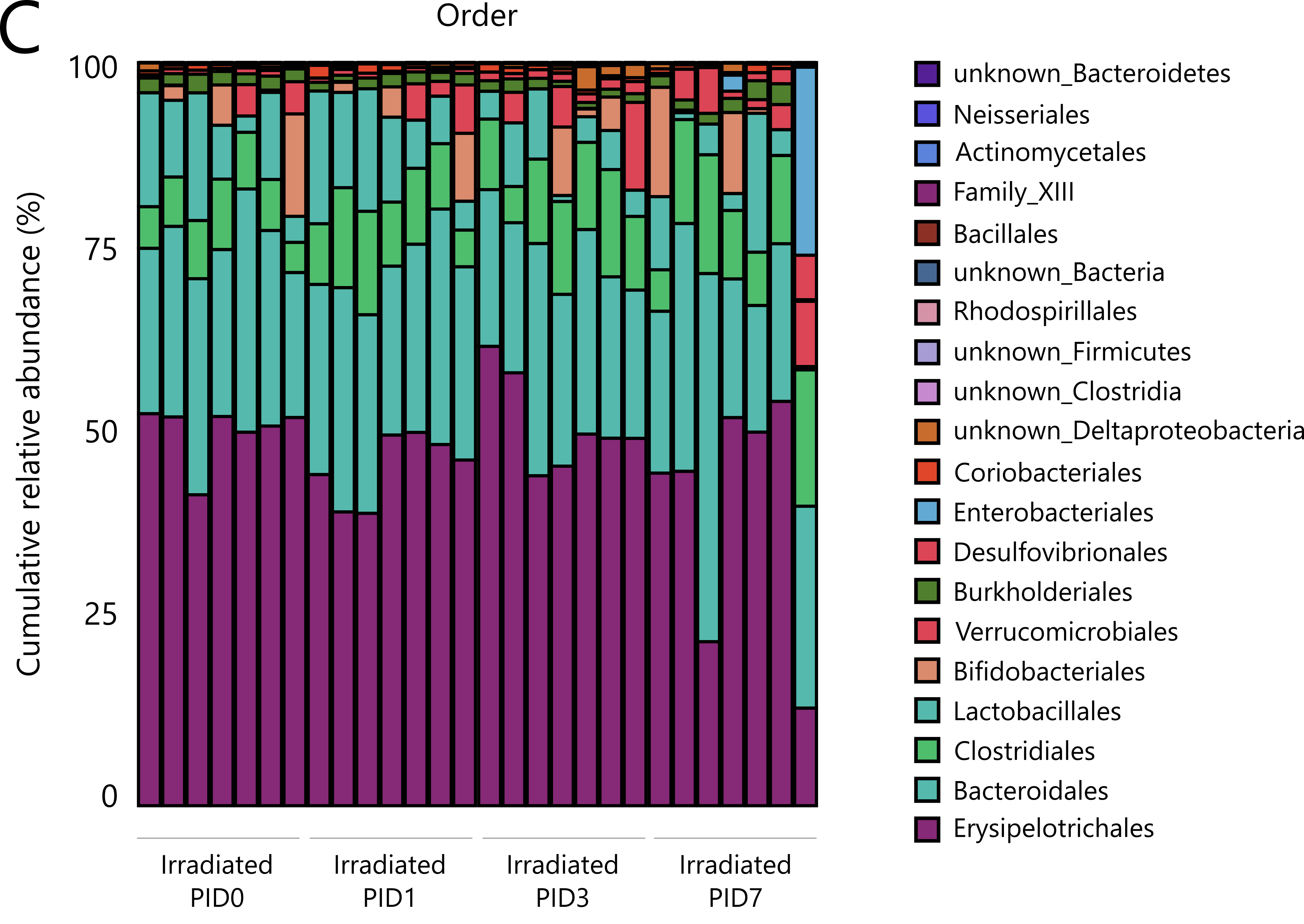


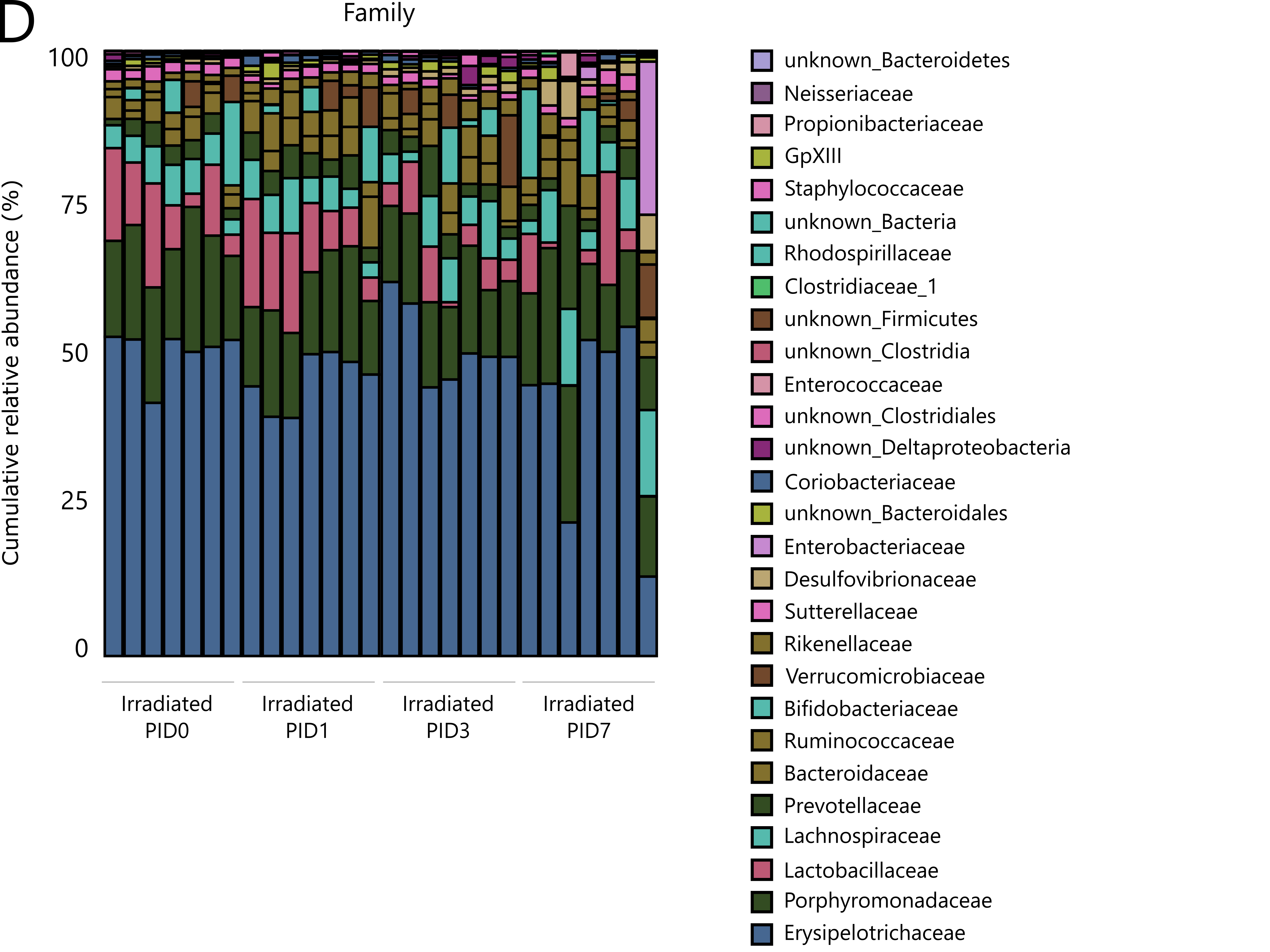


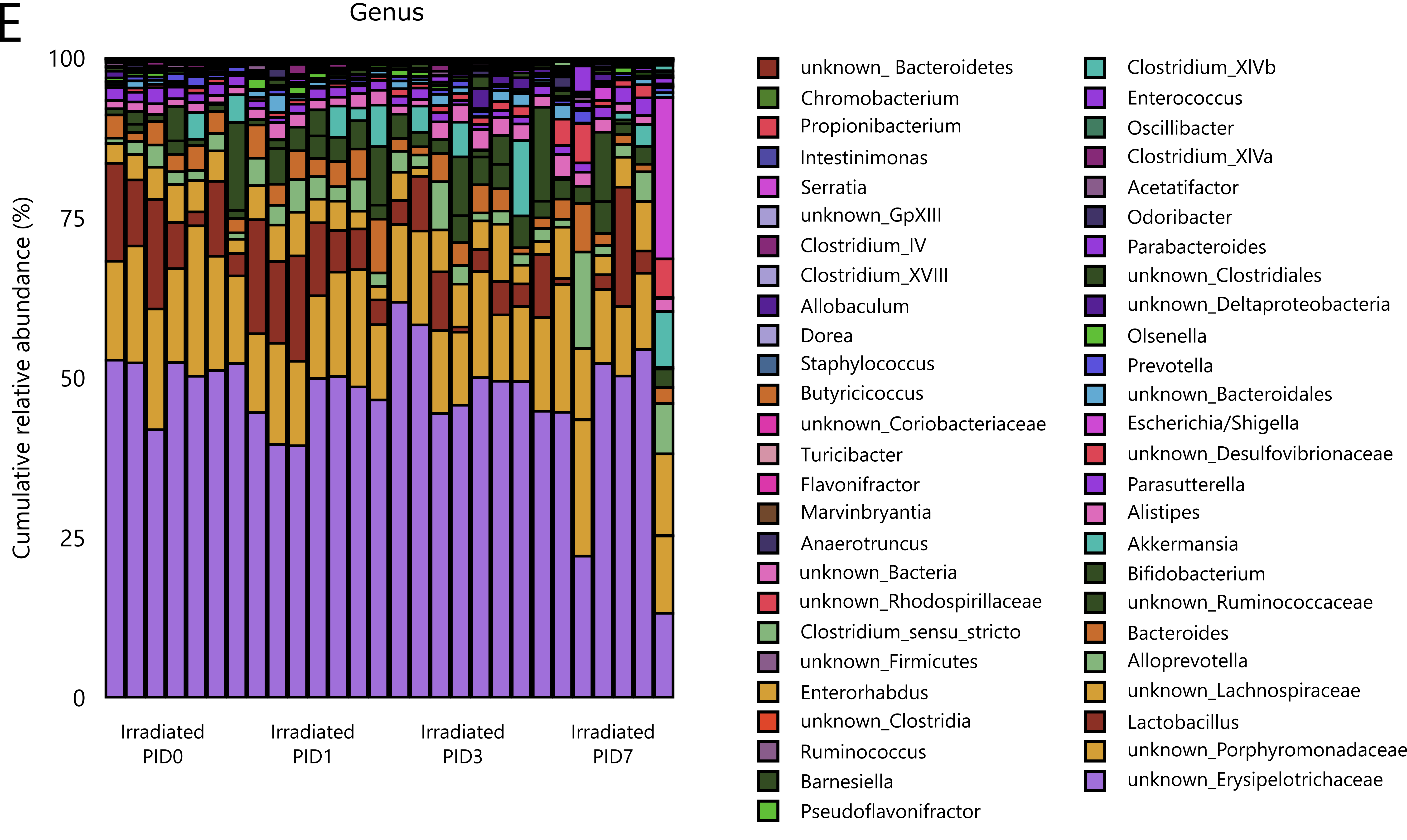


Supplementary figure 4. A-E) Stacked bar plots representing the taxonomic profile evolution over time following pelvic irradiation, at the levels of (A) phylum, (B) class, (C) order, (D) family and (E) genus, n=7 per time point. PID=post-irradiation day

References:

1. Yan K, Chia L, Li X, Ootani A, Su J, Lee J, et al. The intestinal stem cell markers Bmi1 and Lgr5 identify two functionally distinct populations. PNAS 2012; 109: 466–471.

2. Ruyssers NE, Winter BY De, Man JG De, Loukas A. Therapeutic Potential of Helminth Soluble Proteins in TNBS- induced Colitis in Mice. 2009; 15: 491–500.

3. Breugelmans T, Van Spaendonk H, De Man JG, De Schepper HU, Jauregui-Amezaga A, Macken E, et al. In-Depth Study of Transmembrane Mucins in Association with Intestinal Barrier Dysfunction During the Course of T Cell Transfer and DSS-Induced Colitis. J Crohn’s Colitis 2020.
